# Antibiotic-induced alternations in gut microflora are associated with the suppression of immune-related pathways in grass carp (*Ctenopharyngodon idellus*)

**DOI:** 10.3389/fimmu.2022.970125

**Published:** 2022-08-11

**Authors:** Fei Shi, Yao Huang, Mingxuan Yang, Zhijie Lu, Yanan Li, Fanbin Zhan, Li Lin, Zhendong Qin

**Affiliations:** Guangdong Provincial Water Environment and Aquatic Products Security Engineering Technology Research Center, Guangzhou Key Laboratory of Aquatic Animal Diseases and Waterfowl Breeding, College of Animal Sciences and Technology, Zhongkai University of Agriculture and Engineering, Guangzhou, China

**Keywords:** antibiotics, enrofloxacin, florfenicol, grass carp, gut microbiota, immune response

## Abstract

Gut microbiota play a vital role in fish health homeostasis. Antibiotics are known to alter microbial community composition and diversity; however, the substantial effects of antibiotics upon the gut microbiome with respect to immune-related pathways in healthy fish remain unclear. Accordingly, here we explored the impact of two antibiotics on the intestinal health, immune response, microbiome dynamics, and transcriptome profiles of grass carp. A two-week feeding trial was carried out in which the basal diet was complemented with enrofloxacin (10 mg/kg) or florfenicol (10 mg/kg). The results showed that: (1) Enrofloxacin and florfenicol both induced intestinal oxidative stress and reduced the digestive enzyme activity of grass carp. (2) High-throughput sequencing of 16S rDNA revealed that enrofloxacin but not the florfenicol treatment influenced gut microbiota diversity in grass carp by shifting α/β-diversity with more abundant pathogens detected. (3) Transcriptome profiling demonstrated that florfenicol down-regulated the immune-related pathways of grass carp, and the network analysis revealed that IgA was negatively correlated with certain pathogens, such as *Shewanella* and *Aeromonas*. (4) Antibiotic-induced alternations of gut core microbes were revealed *via* immune-related transcripts, as were lower mRNA expression levels of mucosal-related genes. (5) Apoptosis and histopathological changes were detected in the enrofloxacin- and florfenicol-treated groups compared with the control group. Overall, administering antibiotics will promote oxidative stress, cause intestinal flora dysbiosis, inhibit the mucosal immune system, and induce apoptosis in grass carp.

## Introduction

Aquaculture has rapidly expanded to become an important component of global fish production, playing a significant economic role in the farmed-food industry and providing a stable supply of protein for human consumption. However, more aquaculture production causes environmental pollution and increases the likelihood of disease epidemics. Various pathogenic diseases have caused substantial death and economic losses within the aquaculture industry (1). Because antibiotics have been widely used to prevent severe losses due to infectious fishery diseases, the liberal application and overuse of antibiotics has led to the spread and evolution of bacterial resistance, food safety hazards, and environmental issues (2). Therefore, the occurrence and potential impact of antibiotics are receiving mounting attention worldwide (3, 4).

The use of some antibiotics, including florfenicol and enrofloxacin, is allowed in aquaculture in China. Enrofloxacin, a member of the fluoroquinolone family, is a synthetic chemotherapeutic agent effective against a broad spectrum of gram-negative and gram-positive pathogens in aquatic animals (5, 6). Its anti-bacterial mechanism might function by inhibiting the DNA gyrase enzyme and preventing the supercoiling and synthesis of DNA (7). Mounting evidence is showing that enrofloxacin induces oxidative stress, cell apoptosis, and metabolic disorders in fish, thus posing tremendous threats to the host species under farming (6, 8, 9). Florfenicol, a fluorinated derivative of thiamphenicol, is considered an efficient, broad-spectrum antimicrobial substance that inhibits transpeptidation during protein synthesis in bacteria, rendering it effective against many gram-negative and gram-positive bacteria (10). Many studies have reported on the pharmacokinetics and eco-toxicological effects of florfenicol on various fish species (11–14). Both antibiotics might become main sources of pollution by entering the aquatic environment *via* the disposal of unused drugs or the excretion of non-metabolized derivatives in hosts. Consequently, due to its high-water solubility, low bioavailability, and widespread misuse, how enrofloxacin or florfenicol impacts the underlying physiology and health of fish is of great concern but not yet clear.

The gut microbiome of aquatic fish, densely colonized by diverse and large populations of microbial species, plays a vital role in the defense mechanisms of the host by influencing its nutrient digestion, immune system modulation, and resistance to intrusive pathogens (15–17). Compared with that of mammals, the gut microbiota of fish varies more among individuals and is more likely changed by extrinsic environmental factors, such as abiotic stress as well as antibiotics. Studies have found that the antibiotic-treated gut microbiome induces a fragile dysbiosis by reducing bacterial diversity (13, 18). Although the fish gut is the main vehicle for antibiotic absorption, most studies have focused on the gills, liver or kidney as targeted sites when evaluating the risks posed by antibiotics (19, 20). Additionally, exposure to antibiotics is known to cause short-term or long-term variation in the gut microbiome (13, 21, 22), yet little is known about the impact of oral antibiotic treatments upon the gut microbiota of fish. Moreover, there is a general lack of understanding of their effects on fish immune function and metabolic pathways.

Grass carp (*Ctenopharyngodon idellus*) is now one of the most abundantly farmed fish worldwide. However, cultured grass carp populations in China incur serious disease problems caused by pathogenic infections, which is impeding the sustainability of industry development and generating significant economic losses. Accordingly, to better address the concerns and questions associated with the impact of administered enrofloxacin and florfenicol to grass carp, here we investigated the immune response, bacterial diversity, and transcriptome of the intestine of *C*. *idella* treated with these oral antibiotics. The aim of this study was to provide an in-depth evaluation of the antibiotic-induced patterns and dynamics of the microbiota grass carp and the potential mechanism involved.

## Materials and methods

### Experimental design and sample collection

Grass carp individuals (*C*. *idella*, approximately 64 g each) were obtained from fish farm sites in Guangzhou city (Guangdong Province, China). To acclimate the fish, they were placed in tanks with clear and bubbling fresh water at a 28 ± 2°C temperature for 1 week prior to the experiments. The antibiotics enrofloxacin (CAS Number: 93106-60-6) and florfenicol (CAS Number: 73231-34-2) were purchased from Aladdin (Shanghai, China). The fish were unsystematically divided into three groups of 270 fish with triplicates. The corresponding fish dietary treatments were prepared using a basic feed (purchased from Tongwei Group, Chengdu, China) for the control group (CG) and the same but supplemented with enrofloxacin (EG) or florfenicol (FG) at a concentration of 10 mg/kg. After undergoing a 2-week feeding trial, all fish were starved for 24 h and then anaesthetized with tricaine methanesulfonate (MS-222). The midgut tissues of 12 individual fish from each of the three treatment groups were snap-frozen and aseptically harvested in liquid nitrogen for their biochemical, microbial, and transcriptomic analyses.

### Biochemical assays

The midguts of six grass carp fish per tank were homogenized with 0.8% sterilized saline solution and then centrifuged. The ensuing supernatant was measured for biochemical parameters in a microplate reader (Bio-Rad, USA). Catalase (CAT, A007-1-1), total superoxide dismutase (T-SOD, A001-1-1), amylase (AMS, C016-1-1) and lipase (LPS, A054-1-1) activities were quantified by using appropriate commercial assay kits of the Nanjing Jiancheng Institute (China) and following the manufacturer’s protocol.

### DNA extraction, high-throughput sequencing, and bioinformatic analyses of gut microbiota

The grass carp midguts were aseptically dissected and collected from the control, enrofloxacin, and florfenicol groups (respectively, n = 12 fish per tank). Total genomic DNA was isolated from the midgut samples with a DNeasy PowerSoil kit (Qiagen, Hilden, Germany) according to its manufacturer’s instructions. DNA concentration and integrity were respectively checked using a NanoDrop 2000 spectrophotometer and agarose gel electrophoresis. The V3–V4 hypervariable region of 16S rDNA was amplified by a universal pair of primers (343F-798R; **Table 1** **)**. Sequencing libraries were created by blending the amplicons and then assembling them on Illumina MiSeq platform PE300, this done externally by the OE Biotech Co. Shanghai, China.

**Table 1:** Primer sequences for real-time PCR analysis.

Gene	Primer	Sequence (5’- 3’)
***MHC2***	Forward	CGACTTCTACCCTCAACC
	Reverse	GGCGTGCTCCACCATACA
***IgA***	Forward	GGTGTTTACTGTGTTGAGCTCAC
	Reverse	TACCACCACTGCAAAGATATCCC
***Igα***	Forward	GAACTGAGCAAGAAGAAAGGACG
	Reverse	GACCCTGTGGATGGATATGTGAA
***Igβ***	Forward	GACTGAACAAAGAAATGCCTCCC
	Reverse	CAGGCTAGCGAAGAAAGAAAGTG
***Card11***	Forward	ATGAGGACTGGCTGTTCTAAGTG
	Reverse	AGAGGGCTGACTGTGATAAGTTG
***Fcrl5***	Forward	TACCTGGGTTTACTTCCTTGAGC
	Reverse	TTCCATTGACAGATTCTCCTGGG
***PI3K***	Forward	AGAAGGAGGAGGAGTGGTATGAA
	Reverse	GCGAATTCTCTCCTCAGAGGTAG
***Itgb2***	Forward	AATGAGAGAGGTAGGTGAGTCCA
	Reverse	GAAACGTTATAATCCCCTGCGTG
***Claudin6***	Forward	CATCGGGAACAACATTGTAACGG
	Reverse	GTACAGATGAGCAAAGCGAGAAC
***Occludin***	Forward	GCAGTACGGATTAGGTTACGGAT
	Reverse	GTAAGGGGTCGGGTAAGAATTGT
***ZO-1***	Forward	GTGAAAACACCCAGTGACATCAG
	Reverse	CAATCTCCCAGGTGTCTAAAGCT
***ZO-2***	Forward	GTGTTTCAAGTGTGGGTATCTGC
	Reverse	GTTGTAACAGGTGTGTCGAAAGG
***ZO-3***	Forward	AAGCACCTAACCCTGGATGTAAG
	Reverse	AGATTACCTCTGGGATCAGGTGA
***β-actin***	Forward	ACCCACACCGTGCCCATCTA
	Reverse	CGGACAATTTCTCTTTCGGCTG
***343F***	Forward	TACGGRAGGCAGCAG
***798R***	Reverse	AGGGTATCTAATCCT

Paired-end tags were merged using FLASH tool (v1.2.7) to obtain the raw sequences. Parameters with an average quality score < 20 in a 50-bp sliding window were filtered using Trimmomatic, and then sequences < 350 bp and any predicted possible chimeras eliminated by UCHIME. After removing low-quality reads, the credible ones were clustered into operational taxonomic unites (OTUs) by USEARCH (v10.0) at 97% similarity levels. The representative sequence for each OTU was extracted by the QIIME package. A table for total OTUs was standardized to a depth of 35 014 tags per sample. All amplified sequences were submitted for comparison against the SILVA database (v138) through Ribosomal Database Project (RDP) classifier (using an 80% confidence threshold).

The bioinformatic analyses were performed following our recent study albeit with slight modifications (23). Briefly, the bacterial diversity of each midgut sample was estimated in terms of α-diversity, as the Simpson index and Shannon index. Rarefaction curves were constructed to verify that high-through sequencing reached saturation. For the β-diversity analysis, Bray-Curtis and unweighted-UniFrac distance matrix methods were applied for the principal coordinates analysis (PCoA), by using QIIME package. The taxonomy bars, heatmap, and co-occurring species network visualization were obtained in QIIME and drawn in R software.

### RNA extraction, cDNA synthesis, and transcriptomic sequencing analyses

To reveal the immune-related and metabolic-related gene regulation induced by antibiotics in the intestines of grass carp, transcriptomic analyses were processed according to our recent study (24). Total RNA was extracted from the midgut of grass carp by using the TRlzol Reagent and following the manufacturer’s instructions (Life Technologies, California, USA). Next, the cDNA libraries were constructed as per the instruction manual of NEBNext Ultra RNA Library Prep Kit. Finally, all these built cDNA libraries were sequenced on an Illumina Hi-Seq X Ten system by the OE Biotech Co., Ltd. (Shanghai, China).

Low-quality reads with unknown nucleotides of > 5% or a Q20 < 20% were filtered out using Trimmomatic software. After removing these low-quality sequences, the remaining clean reads were separately aligned as per the *C*. *idella* genome by Tophat2 software. Gene expression levels were calculated as fragments per kilobase of exon per million fragments mapped (FPKM) values, by Cufflinks software. To assess differentially expressed genes (DEGs) and q-values for CG, EG, and FG, two parameters (a false discovery rate [FDR] significance score < 0.01 and logarithmic fold ratio ≥2) were threshold criteria for inclusion in subsequent analyses. Gene Ontology (GO) and Kyoto Encyclopedia of Genes and Genomes (KEGG) pathway enrichment analyses of designated DEGs were implemented using the Goatools and KOBAS packages, respectively.

### Expression of immune-related and tight junction protein genes by qRT-PCR

Total RNAs were extracted from the midguts of grass carp in the CG, EG, and FG by applying the TRIzol reagent (Sangon, Shanghai). The cDNA libraries were constructed by using the PrimeScript™ RT reagent kit combined with gDNA Eraser according to the manufacturers’ protocols. For the validation of RNA-seq data, eight DEGs—major histocompatibility complex2 (*MHC2*), immunoglobulin A (*IgA*), B-cell antigen receptor complex-associated protein alpha/beta chain (*Igα*/*Igβ*), caspase recruitment domain-containing protein 11 (*Card11*), Fc receptor-like protein 5 (*Fcrl5*), phosphatidylinositol 4,5-bisphosphate 3-kinase catalytic subunit (*PI3K*), and integrin beta-2 (*Itgb2*)—active in the intestinal immune network with respect IgA production, B cell receptor signaling, and phagosome pathways were chosen to undergo quantitative real-time PCR (qRT-PCR).

Moreover, the relative mRNA expression levels of several tight junction protein genes, namely *Claudin6*, *Occludin*, and those encoding zonula occludens (*ZO-1*, *ZO-2*, and *ZO-3*) were also determined. The β-actin gene served as the internal control and the primers for the above genes are listed in **Table 1**. Further qRT-PCR experimental procedures were followed as describe in our recent work (25). Briefly, the ChamQTM SYBR^®^ qPCR Master Mix (Vazyme, Nanjing, China) was used for qRT-PCR run under the following conditions (in triplicate): 95°C for 30 s followed by 40 cycles of 95°C for 10 s, 60°C for 30 s, and 72°C for 20 s, then ending with a final incubation at 4°C for 5 min. The relative expression level of each selected gene versus β-actin gene was calculated according to the 2^-ΔΔCT^ method.

### Correlations between transcript variables and gut microbes

For the three different treatment groups, we chose eight immune-related transcripts (*MHC2*, *IgA*, *Igα*, *Igβ*, *Card11*, *Fcrl5*, *PI3K*, and *Itgb2*) as the response variables and top-ranked 30 genera of the midgut microbiome as the other set of variables to test for significant correlations between targeted immune transcripts and core genera. Network coefficients were calculated using ‘igraph’ and ‘network’ packages in R software. Spearman’s rank test was used with statistical significance ascribed to a p-value ≤ 0.05 and q value ≤ 0.1. Only the significant correlations were included in the network and its visualization in which each core genus or immune-related gene is represented by a node and their positive or negative correlation indicated by their shared edge. The networks were drawn using Cytoscape software v3.7.2.

Redundancy analysis (RDA) was applied to detect and convey correlations arising between core midgut microbes at the phylum level and the variables of tight junction proteins among the three treatment groups. Top-ranked 10 genera and tight junction proteins (*Claudin6*, *Occludin*, *ZO-1*, *ZO-2*, and *ZO-3*) were used as the inputs and RDA performed using the ‘vegan’ package in R software.

### Gut morphology

After the feeding period, six grass carp fish from each treatment group were randomly chosen and anaesthetized with tricaine methane sulfonate (MS 222). Their intestines were fixed separately with 4% paraformaldehyde for 24 h and placed in ethanol under a series of concentrations for their dehydration. Afterwards, the collected tissue samples were processed and embedded in paraffin wax. The midguts were exposed and stained with hematoxylin-eosin (H&E). The target-stained slides were observed and digitally photographed under a Canon PowerShot G6 with 7.1 megapixels (Canon 219 Inc., Tokyo, Japan).

### Apoptosis analysis

To evaluate apoptosis, the TdT-mediated dUTP nick-end labeling (TUNEL) staining assay was used. It was performed using a one-step TUNEL apoptosis assay kit (Guge Biology, Wuhan, China) applied to the fixed midgut tissues. The cell nucleus was stained with the DAPI solution (Servicebio, Wuhan, China), at 37°C, for 10 min. The intestinal images were acquired under a fluorescence microscope (Leica DMI8, Wetzlar, Germany).

### Statistical analysis

The mean ± standard deviation (SD) values were obtained from the data and collated accordingly. Significant differences among the three treatment groups (CG, EG, and FG) were examined using one-way analysis of variance (ANOVA) followed by Dunnett’s *post hoc* test. PCoA significance for the Bray-Curtis and unweighted-UniFrac distance matrix was tested with the analysis of similarities (ANOSIM) to compare taxa in the control (CG) with those in the antibiotic treatments (EG, FG). All statistical analyses were performed using R (‘vegan’ package). The p < 0.05 value was deemed as statistically significant. The statistical parameter of the KEGG enrichment was set to a Bonferroni-corrected p-value < 0.05. The histograms were derived and drawn using Graph Pad Prism 9.0 (San Diego, CA, USA).

## Results

### Effects of antibiotics on biochemical activity in the midgut of grass carp

**Figure 1:**
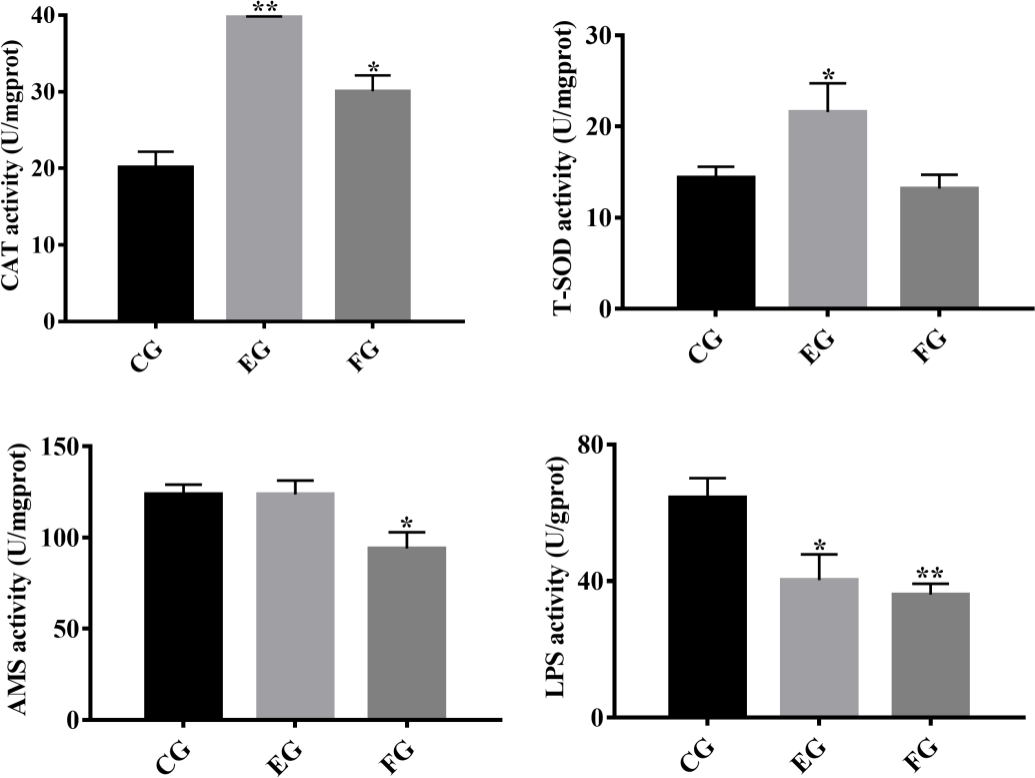
Antioxidative and digestive enzyme indices in the midguts of grass carp fed with different treatments (control (CG), enrofloxacin (EG) and florfenicol (FG)) after two weeks. Catalase (CAT), total superoxide dismutase (T-SOD), amylase (AMS) and lipase (LPS) were selected, and the bars show the mean ± SD (n = 3). The different symbols indicate significant differences (*, p < 0.05; **, p < 0.01) among the groups.

To determine the influence of antibiotics on antioxidant and digestive enzyme activity in the midgut of grass carp, the respective activity of CAT, T-SOD, AMS and LPS was quantified in each treatment group (**Figure 1**). Compared with the control, the antioxidant enzyme CAT’s activity was significantly increased in fish exposed to enrofloxacin and florfenicol. However, antioxidant enzyme T-SOD’s activity was increased significantly by enrofloxacin only. Moreover, grass carp fed with the florfenicol additive had a significant lower activity of the digestive enzymes AMS and LPS, whereas the latter was only significantly reduced when adding enrofloxacin, vis-à-vis the control group.

### Enrofloxacin affects the diversity of midgut microbiota in grass carp

**Figure 2:**
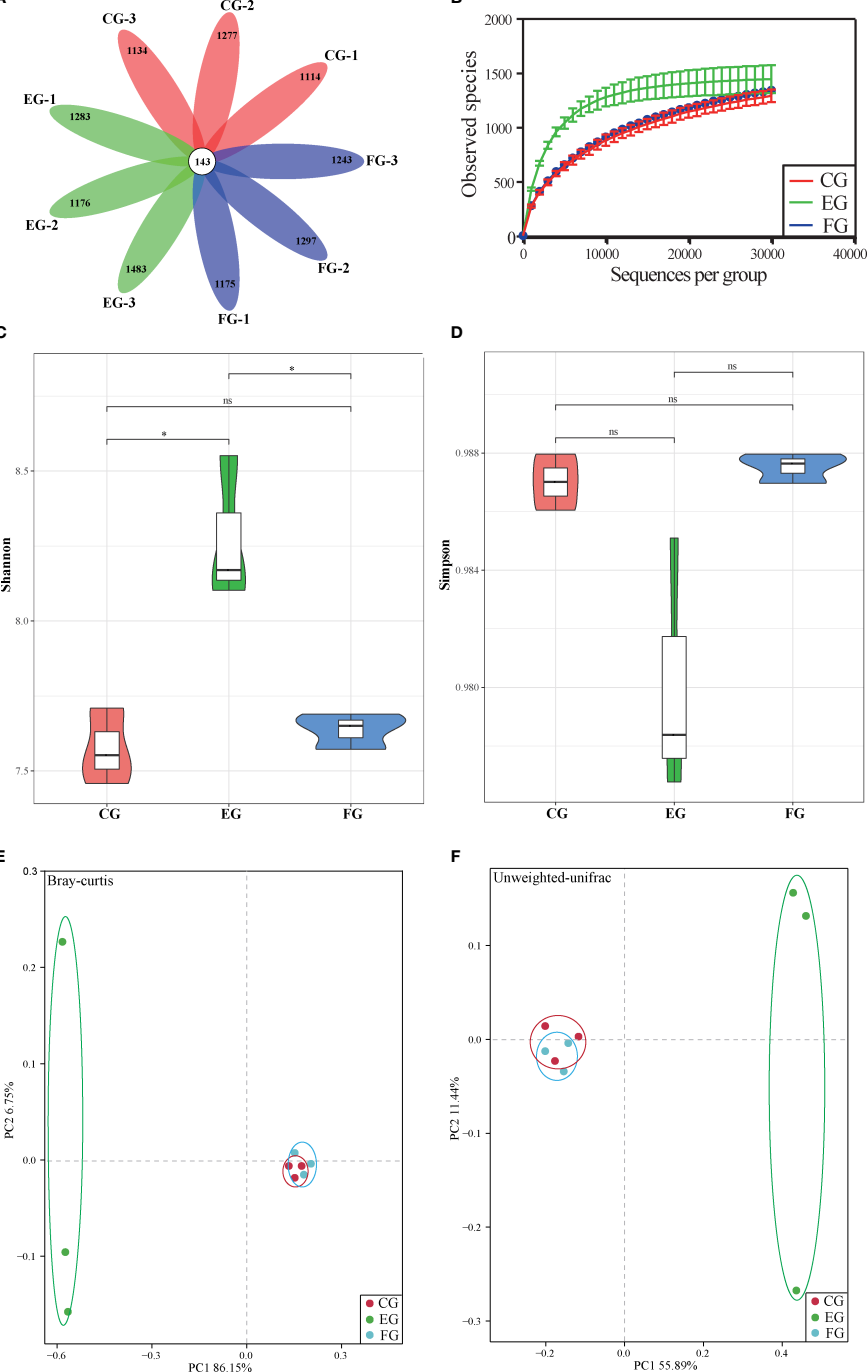
Alpha-diversity and beta-diversity of midgut bacterial community of grass carp with different diets (control group (CG), enrofloxacin group (EG) and florfenicol group (FG)). **(A)** Venn diagram of shared and unique OTU numbers per sample. **(B)** Rarefaction curves of OTUs clustered at 97% sequence similarity across different groups. Bars show the mean ± SE (n=3). The different symbols represent significant differences (*, p < 0.05; ns, no significant). **(C)** Shannon-Wiener (Shannon) index. **(D)** Simpson index. **(D–E)** PCoA of the samples among different treatments ((control, enrofloxacin and florfenicol) using bray-Curtis and unweighted-unifrac method.

After discarding the low-quality reads, the final number of valid OTUs in each group was about 1257~1626. A total of 143 OTUs were common to the control and antibiotic-treated groups (with three duplicates) in the Venn diagram analysis, while the number of unique OTUs was similar among the three groups (**Figure 2A**). A tendency toward saturation was observed in rarefaction curves, illustrating relatively sufficient and robust sequencing depth (**Figure 2B**). The α-diversity values for Shannon and Simpson indexes are shown in **Figures 2C, D**. The Shannon index was significantly higher for the midgut of the enrofloxacin group than the control group; however, no significant difference was observed between florfenicol group and control group (**Figure 2C**). Unlike the Shannon index, the Simpson index did not differ significantly among the three treatments (**Figure 2D**). From the PCoA based on Bray-Curtis (**Figure 2E**) and unweighted-UniFrac (**Figure 2F**) distance matrices, whose results showed the same patterns, it is evident that the florfenicol and control groups’ communities clustered together; conversely, the enrofloxacin communities were well separated from those. According to the Bray-Curtis and unweighted-UniFrac distance methods, notable ANOSIM differences were significantly detected between the control and enrofloxacin groups (p < 0.05) but none between the control and florfenicol groups (p > 0.05).

### Enrofloxacin modulates major and specialized taxa in grass carp’s midgut

**Figure 3:**
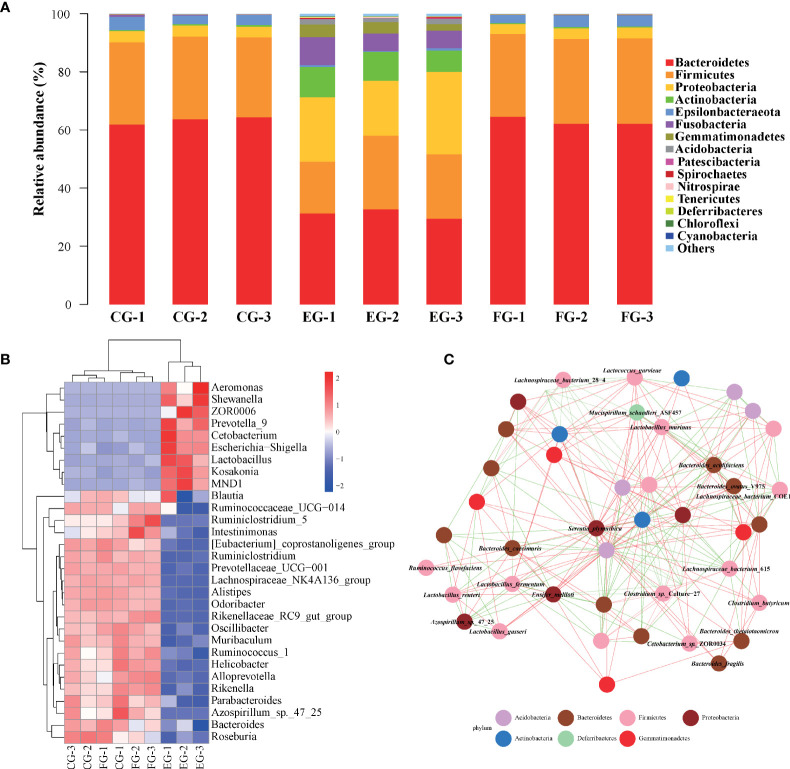
Major dominant and specialized targets of bacterial communities in different antibiotic-treated groups. **(A)** Relative taxonomic abundance of bacterial communities at the phylum level found in different groups. **(B)** Heatmap of relative abundances of the 30 most dominant genera summarized in the midgut microbial communities from three different groups. **(C)** Co-occurring species network according to correlation analysis using top 50 species with Spearman coefficient > 0.7 and p value < 0.01. Red and green edges represent positive and negative correlations, respectively. The phylum taxonomy is marked through the node colors.

To comprehensively reveal the detailed composition of the midgut microbial community in antibiotic-treated grass carp, the relative abundances of bacteria at the phylum level were examined (**Figure 3A** **)**. According to the taxonomic identification, Bacteroidetes, Firmicutes, and Proteobacteria were the most dominant phyla in all treatment groups. In comparison with the enrofloxacin group, more similar microbial composition patterns, such as the relative abundances of Bacteroidetes and Firmicutes being over 80%, were found between the control and florfenicol groups. The phylum Epsilonbacteraeota was relatively more abundant in the control and the florfenicol groups though barely present in the enrofloxacin group. The bacterial community in the enrofloxacin group was more diverse, having a lower abundance of Bacteroidetes than in the other two groups. Additionally, relatively higher proportions for the phyla of Proteobacteria, Actinobacteria, Fusobacteria, and Gemmatimonadetes were detected in the enrofloxacin group.

A heatmap (**Figure 3B**) shows the relative composition profile of top-30 prevailing genera in the three treatment groups. Evidently, the genus *Bacteroides* was the most abundant across the groups. Significant differences in the relative abundances of some genera were also detected between the control and antibiotic-treated groups according to **Figure 3B**. For instance, the dominance of *Bacteroides*, *Helicobacter*, and *Odoribacter* was greater in the control and florfenicol groups; however, some other genera, such as *ZOR0006*, *Cetobacterium*, *Escherichia-Shigella*, *Shewanella*, *MND1* and *Lactobacillus* were more abundant in the enrofloxacin group.

The co-occurring species network (**Figure 3C**) for the top-50 ranked abundant bacterial species across all the groups was inferred to illustrate the discriminatory core taxa in the grass carp midgut. Positive or negative interactions between species were found, with any taxa classified as uncultured not shown and likewise for ambiguous bacteria. *Serratia plymuthica* and *Bacteroides acidifaciens* were the relatively frequent species associated with some other taxa. Meanwhile, some probiotic species such as *Clostridium butyricum*, *Lactobacillus gasseri*, and *Lactobacillus murinus* were also present (**Figure 3C** **)**.

### Florfenicol alters the gut transcriptome profiles in grass carp

**Figure 4:**
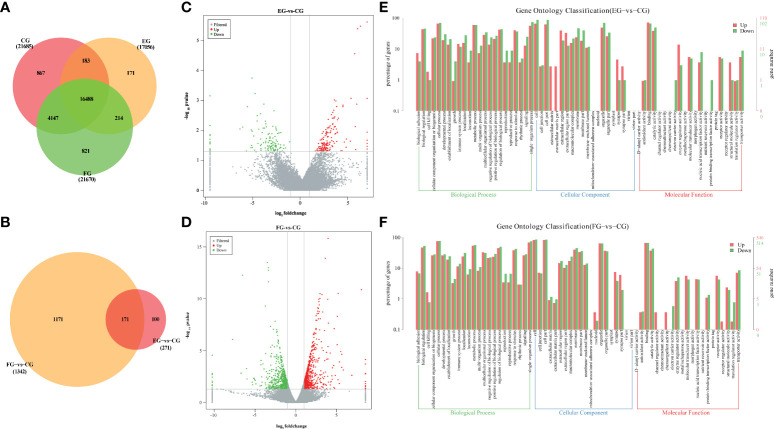
Antibiotics modulated genes expression in the intestine of grass carp. **(A)** Venn diagram of annotated genes in different groups (CG, EG and FG). **(B)** Venn diagram of DEGs in different antibiotic diets groups compared with control group (EG-*vs*-CG and FG-*vs*-CG). **(C, D)** Volcano plots of DEGs between the enrofloxacin group **(C)** and florfenicol group **(D)** compared with control group, respectively. **(E, F)** Gene ontology (GO) classification in second level of the unigenes between the enrofloxacin group **(E)** and florfenicol group **(F)** compared with control group, respectively.

To elucidate the fundamental mechanism(s) responsible for the effects of antibiotics on gene expression levels in the midgut of grass carp, transcriptomic profiling of control vis-à-vis antibiotic-treated groups was performed. After the mRNA sequencing, a total of 65.18 Gb of clean data was obtained from all three groups, consisting of 6.97~8.12 Gb per sample. A total of 24 413 known genes and 246 novel genes were functionally annotated (data not shown), of which 16 488 genes were shared among three groups (**Figure 4A**). Compared with the control group, 271 and 1342 DEGs were identified only in the enrofloxacin group and florfenicol group, respectively (**Figure 4B**). The volcano plot for control versus enrofloxacin group included 150 up-regulated and 121 down-regulated genes (**Figure 4C**). By contrast, 705 up-regulated and 637 down-regulated genes were identified between the control and florfenicol groups (**Figure 4D**).

According to the GO analysis, similar patterns characterized the enrofloxacin and florfenicol groups when compared to the control group, for which a total 64 significant enrichment records were screened, corresponding to 23 cellular components, 20 biological processes, and 21 molecular functions (**Figures 4E, F** **)**. Moreover, the terms “single-organism process” under biological processes, “cell part” under cellular components and “binding” under molecular functions were the most well-represented by the GO annotations in each main category, respectively. Nevertheless, more up-regulated DEGs were distinguished for “protein binding transcription factor activity” under molecular functions in the florfenicol group than enrofloxacin group compared with the control group.

**Figure 5:**
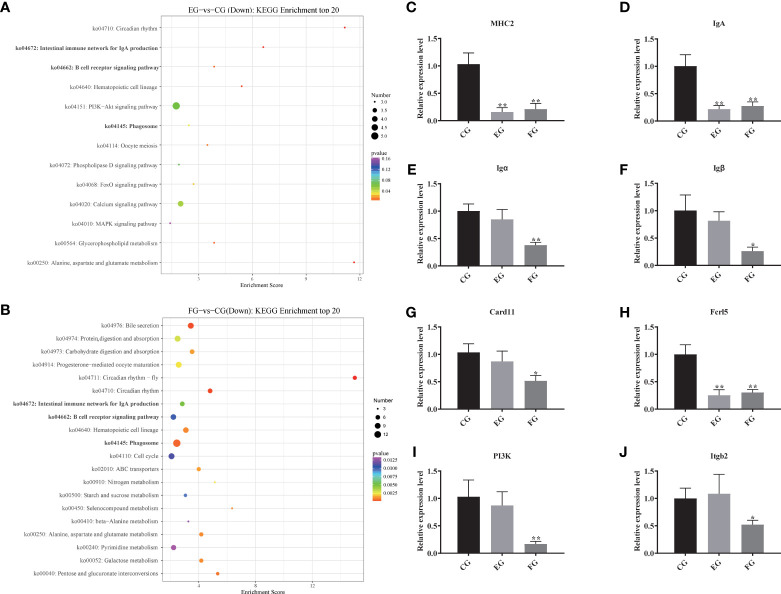
Pathway enrichments of all DEGs related to immune function. **(A, B)** The most enriched KEGG pathways of all DEGs in response to antibiotic groups in the intestine of grass carp compared with the control group. **(C–J)** Immune genes expression levels of *MHC2*, *IgA*, *Igα*, *Igβ*, *Card11*, *Fcrl5*, *PI3K* and *Itgb2* mRNA in the intestine of grass carp fed with the control and antibiotic diets for 2 weeks. The different symbols show significant differences (*, p < 0.05; **, p < 0.01).

Compared with the control group, the top-20 KEGG downgraded pathway analyses, that is those whose q-values were lowest, were summarized for enrofloxacin (**Figure 5A**) and florfenicol (**Figure 5B**) groups. More specifically, in both comparisons, the shared KEGG enrichment pathways were closely involved in immune functioning processes, including the intestinal immune network for IgA production, the B cell receptor signaling pathway, and the phagosome.

To evaluate the DEG library with respect to the immune pathways mentioned above, eight pathway-related DEGs were assessed *via* qRT-PCR (**Figures 5C–J**). The relative expression levels were significantly decreased for most DEGs, especially *MHC2*, *IgA*, and *Fcrl5*, in the enrofloxacin and florfenicol groups when compared to the control group. However, the DEGs *Igα*, *Igβ*, *Card11*, *P13K*, and *Itgb2* showed no significant differences between the control and enrofloxacin groups.

### Association between gut microbes and immune-related transcripts

**Figure 6:**
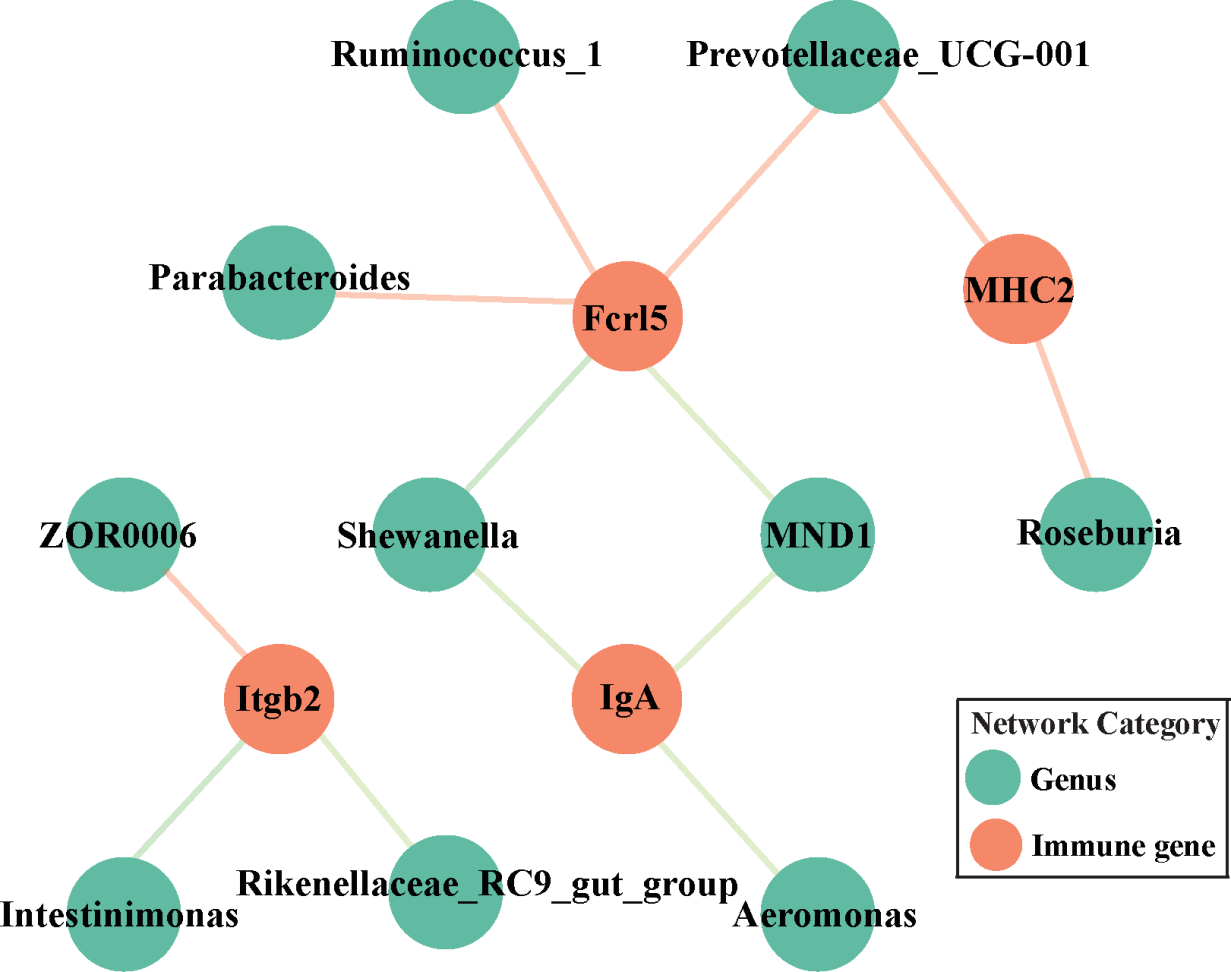
Network analysis of the correlation between targeted genera and discriminatory immune-related genes (*IgA*, *MHC2*, *Fcrl5* and *Itgb2*) among all the groups. Orange node is mapped to immune-related genes while green one represents targeted core genera. Pink and light green edges indicate positive and negative correlations, respectively.

To clarify the relationships between the midgut microbiome and the targeted immune pathways in grass carp, a correlation network was generated (**Figure 6** **)**. This indicated only four DEGs significantly linked to the core bacterial genera. Intriguingly, *IgA* was always negatively correlated with *Shewanella*, *Aeromonas*, and *MND1*; although *Fcrl5* was negatively correlated with *Shewanella*, it was positively correlated with *Parabacteroides*, *Ruminoccoccus_1*, and *Prevotellaceae_UCG-001*. *MHC2* was positively correlated with *Prevotellaceae_UCG-001* and *Roseburia*. Consistently, *Itgb2* was positively correlated with *ZOR0006* yet negatively correlated with *Rikenellaceae_RC9_gut_group* and *Intestinimonas*.

### Tight junction proteins associated with key microbial genera

**Figure 7:**
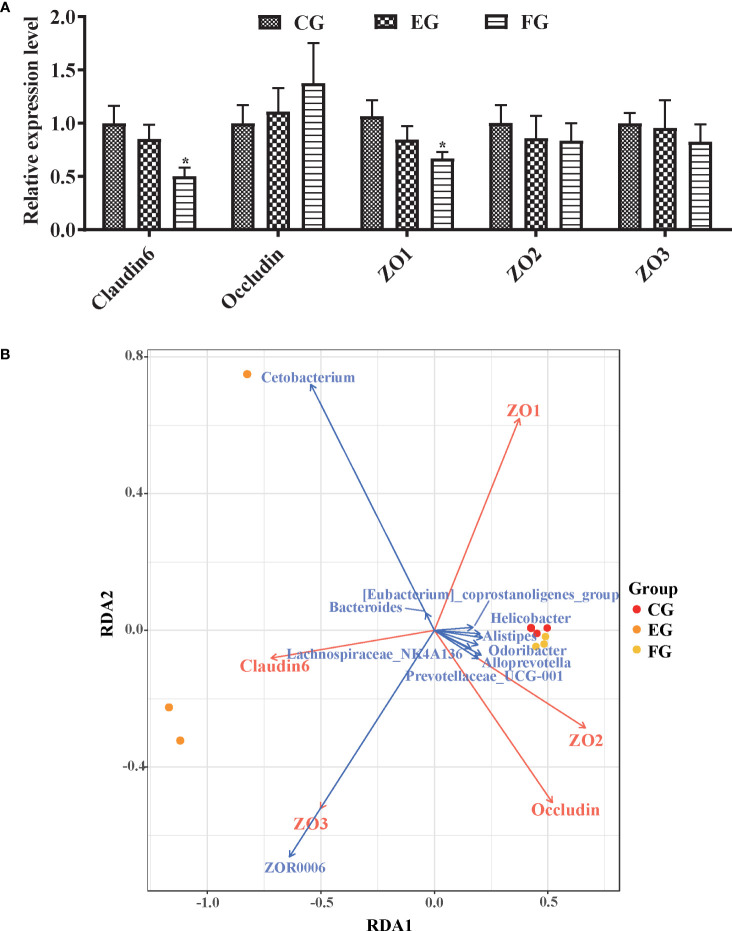
Relative expression levels of tight junction protein genes associated with the core microbiomes. **(A)** Immune genes expression level of tight junction proteins *Claudin6*, *Occludin*, *ZO-1*, *ZO-2*, and *ZO-3* mRNA in the midgut of grass carp fed with the control and antibiotic diets for 2 weeks *via* qRT-PCR. The different symbols show significant differences (*, p < 0.05). **(B)** RDA profile of the correlation between tight junction proteins (red arrows) and key genera (blue arrows) in the midgut of grass carp fed with control, enrofloxacin and florfenicol groups.

The respective impact of the two antibiotics upon the mucosal immune system was inferred by analyzing the mRNA expression of tight junction proteins (*Claudin6*, *Occludin*, *ZO-1*, *ZO-2*, and *ZO-3*) in the midgut of grass carp by qRT-PCR (**Figure 7A**). Interestingly, only the relative expression levels of *Claudin6* and *ZO-1* in the florfenicol group were significantly lower than those of the control group (p < 0.05).

Correlations between the core microbiome and functional genes were assessed *via* RDA (**Figure 7B**). Among all genera, *Cetobacterium* and *ZOR0006* were the major representative taxa for interpreting the microbial communities in the three treatment groups. For instance, *Cetobacterium* was positively correlated with *Claudin6*, as was *ZOR0006* with *ZO3* and *Claudin6*. Likewise, *ZO-1* was the most crucial factor to impact the key genera; for example, *ZO-1* was positively correlated with *Bacteroides* and *Cetobacterium* yet negatively correlated with *ZOR0006.*

### Antibiotics change the intestinal morphology of grass carp

**Figure 8:**
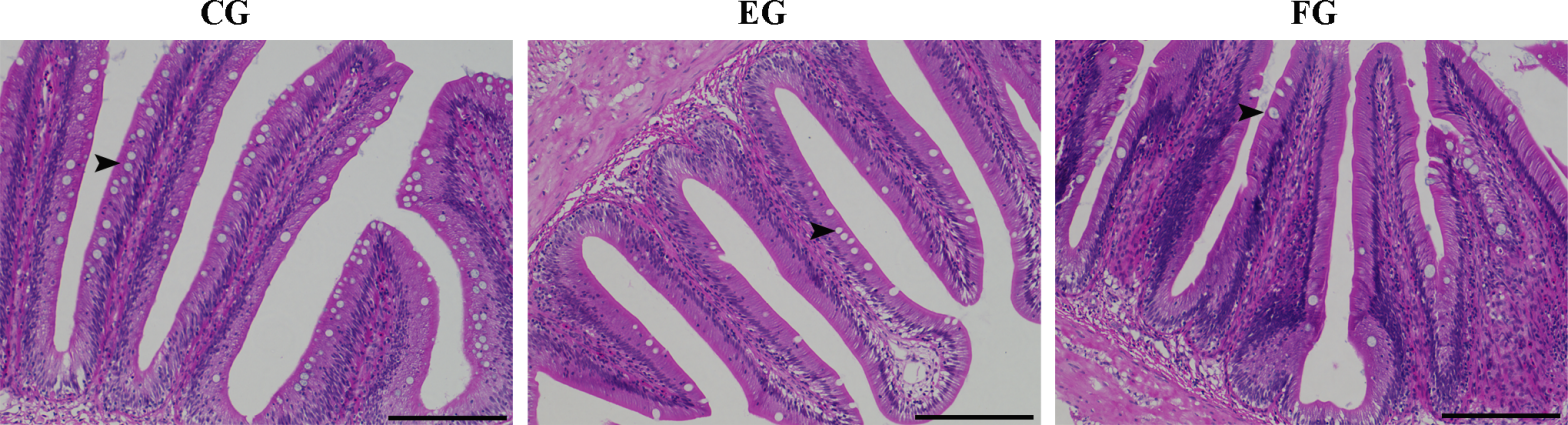
Histopathological changes showing the comparative histology midgut tissues of grass carp fed with different treatments (control (CG), enrofloxacin (EG) and florfenicol (FG)) after two weeks. Representative images of gut tissues were stained by H&E and then analyzed by light microscopy. Arrows referred to goblet cell. Bars are 100 μm.

Midgut tissues of grass carp fish fed for 2 weeks in the control and antibiotic-reduced groups were collect and morphologically analyzed. **Figure 8** are photomicrographs of midgut tissue morphology stained with H&E. As expected, intestinal mucosa of grass carp in the control group appeared relatively normal with no signs of damage or injury. But the goblet cells in the enrofloxacin group were significantly reduced in density, being less abundant in the florfenicol group.

### Antibiotics activated apoptosis in the midgut tissues

**Figure 9:**
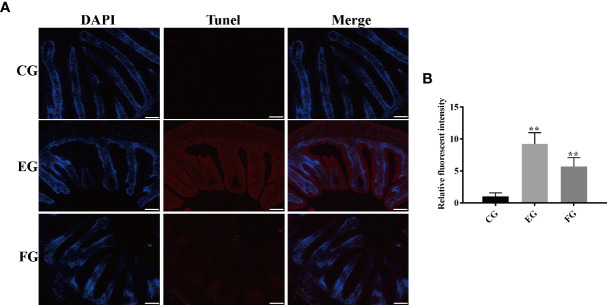
Antibiotics induced apoptosis in the midgut tissues of grass carp fed with different treatments (control (CG), enrofloxacin (EG) and florfenicol (FG)) after two weeks. **(A)** TUNEL staining showing apoptosis in red fluorescence after antibiotic treatments. Scale bar, 100 μm. **(B)** Mean fluorescence intensity based on the TUNEL signal in EG and FG, compared with CG (**p < 0.01).

To further investigate the effect of antibiotics, apoptosis in the midgut of grass carp treated with dietary enrofloxacin and florfenicol for 2 weeks was also assessed. The fluorescence data in **Figure 9A** clearly shows that enrofloxacin or florfenicol markedly increased the TUNEL signal of grass cap midguts; hence, both antibiotics activated apoptosis when compared with the control group. This result was confirmed by the relative fluorescence intensities of the TUNEL signal among all the three groups, showing that either enrofloxacin or florfenicol was capable of significantly inducing apoptosis in the intestines of grass carp fish (**Figure 9B**).

## Discussion

Some antibiotics, including enrofloxacin and florfenicol, are permitted and extensively used in the aquaculture industry, for which most corresponding studies have focused on the distribution, ecology, and evolution of antimicrobial resistance genes in bacterial pathogen communities of fish (26–28). The association of gut microbiota and transcriptome profiles of healthy fish fed with oral antibiotics has yet to comprehensively analyzed and reported on. To fill this knowledge gap, we conducted an in-depth study of grass carp treated or untreated with enrofloxacin or florfenicol.

Antioxidant and digestive enzymes have been evaluated as indicators of oxidative stress and nutrient absorption conditions in aquatic animals (29, 30). CAT and T-SOD, two major antioxidant enzymes, are able to maintain physiological homeostasis in the cellular antioxidant system (24), and increased oxidative stress could be a toxicological effect caused by antibiotics (31, 32). In our study, enrofloxacin and florfenicol treatments induced an increase in CAT in the midgut of grass carp (**Figure 1**). Qiu, Hu (9) concluded that exposure to enrofloxacin significantly disrupts a NF-κB-mediated immune response by inducing the activities of antioxidant enzymes in the primary macrophages of common carp. Similarly, work by Limbu, Zhou (33) revealed that low dosages of oxytetracycline and sulfamethoxazole induced oxidative stress by increasing SOD and malondialdehyde (MDA) enzyme activities in Nile Tilapia. Digestive enzymes, such as AMS and LPS, enable the intestines to digest intrinsic nutrients along with secreted pancreatic enzymes (34). To the best of our knowledge, no study before has empirically quantified the variation in digestive enzyme activities in healthy fish under antibiotic exposure. In so doing, we found that expression levels of LPS activity were lower under the 14-day oral treatments of enrofloxacin and florfenicol (**Figure 1**), which implies antibiotics could attenuate the intestine digestive capacity of the host fish.

Accumulating evidence suggests the functioning of intestinal microbiota in modulating the immune and metabolic systems of the host is closely related to its homeostasis (35). Studies of healthy fish exposure to antibiotics and related host-microbial interactions could be useful to predict the health risk factors and to uncover the underlying protective mechanisms. Recent research concluded that the richness and diversity of gut microbiota in coral trout (*Plectropomus leopardus*) were not affected by enrofloxacin exposure (36). Another investigation found that a 10-day florfenicol treatment reduced the biodiversity of gut bacteria in catfish (37). Contrary to that, in our study the Shannon index of microbial α-diversity in the midgut was increased significantly in grass carp under the oral enrofloxacin treatment (**Figure 2C**); however, the Simpson index was negligibly changed (**Figure 2D**). This may be explained by the so-called “eubiotic” effect of some antibiotics which can positively impact the populations of gut microbiota (38).

The pronounced β-diversity and discrepancy in variation found in gut bacterial composition between the control and enrofloxacin groups suggests that oral administration of enrofloxacin can alter bacterial communities; however, in this respect, the control and florfenicol groups were similar (**Figures 2E, F**, **3**). It is known that low-dose antibiotics could induce dysbiosis by decreasing the abundance of certain intestinal microbiota (39, 40), also called antibiotic-induced microbiome-depletion (AIMD) (41). In their study, Zarrinpar, Chaix (41) learned that luminal Firmicutes and Bacteroidetes species are reduced in the AIMD-treated mice. Both Bacteroidetes and Firmicutes promptly engage in lipid and bile acid metabolism to maintain systemic functions in the host, including its glucose homeostasis (42). This is consistent with our findings that Bacteroidetes and Firmicutes were less abundant in the enrofloxacin-treated group of grass carp fish (**Figure 3A**). According to the heatmap in **Figure 3B**, the abundance of *Cetobacterium* was increased in the enrofloxacin group. Similar conclusions were reached for the gut of *Gambusia affinis* fish exposed to rifampicin (22) and zebrafish exposed to oxytetracycline (13). Notably, some pathogens, such as *Aeromonas*, *Shewanella*, and *Escherichia-Shigella* were more abundant in the enrofloxacin group. Interestingly, the abundance of probiotic *Lactobacillus* was higher in the enrofloxacin group, whose presence might be interpreted as a conferring a protective mechanism to the host, *via* their colonization of the mucosal epithelium and optimization of the gut environment. These findings showed that enrofloxacin is capable of stimulating susceptible genera, thus causing changes to ecological niches. Ng, Aranda-Diaz (43) highlighted that the recovery of intestinal microbes treated with antibiotics in mice depends on the diet (food) and microbial community structure of the host, as well as its surrounding environment. Oscillatory intestinal microbiome dynamics were observed with an early reduction in gram-negative organisms (day 6) and an overall collapse in diversity (day 11), followed by the re-growth of gram-positive species (day 14) after treatment with antibiotics (44). Here, the α/β-diversities showed similar patterns between the control and florfenicol groups (**Figures 2A–F**; **Figures 3A, B**), which points to the sufficient recovery of intestinal microbiome in grass carp treated with florfenicol. According to **Figure 3C**, the relatively dominant species was *B. acidifaciens*. One recent study revealed that *B. acidifaciens* in the gut exerts protective effects against CD95-mediated liver injury (45). Thus, it was speculated that specific species in the fish gut could provide beneficial substances for the host in response to antibiotics in addition to inhibiting certain opportunistic pathogens, as described above.

Gut transcriptome profiles, which are jointly influenced by the immune system in cooperation with the gut microbiome, are crucial for sustaining the host’s homeostasis. Zhang, Li (46) suggested that florfenicol affects some biological functions of *Streptococcus agalactiae* in tilapia, this being closely associated with the reduction of drug accumulation caused by the ATP-binding cassette transporter pathway. To date, however, no transcriptome analysis has been conducted that entailed oral treatments of antibiotics applied to grass carp. We found that many DEGs, as categorized by KEGG pathways, were closely involved in immunity, including those participating in the intestinal immune network for IgA production, B cell receptor signaling pathway, and phagosome (**Figures 5A, B**), which further confirmed that both enrofloxacin and florfenicol are able to activate immune-related pathways. Based on their KEGG analysis, Qiu, Liu (8) illustrated that the metabolic-related steroid biosynthesis pathway was significantly impacted by enrofloxacin and antibiotic cocktails in zebrafish. We verified the expression of eight key genes in the midgut of grass carp to identify putative molecular mechanisms involved in the impact of antibiotics on immune responses (**Figure 5C**). These qRT-PCR results revealed that all examined genes were down-regulated in the florfenicol group, indicating that florfenicol weakens the host immune system by suppressing its related functional pathways.

The gut microbiome is critical for modulating the immune system, affecting absorption of various signaling molecules involved in host immunity (47). Yet the contribution of gut microbial dysbiosis interactions to changed cellular immune function in fish treated with antibiotics remains inconclusive. We investigated the network formed between gut microbiota and discriminatory immune-related genes (**Figure 6**). There is a negative association between *IgA* and pathogens, such as *Shewanella* and *Aeromonas*, indicating that *IgA* is active against some harmful bacteria. Moreover, a decrease in *Prevotellaceae* may cause a declining abundance of short-chain fatty acids (SCFAs) (48). SCFAs are thought to generate an acidic environment in the intestine, which is conducive for stimulating probiotic taxa and suppressing the proliferation of pathogens (49). We found that *Prevotellaceae* was positively associated with *MHC2* and *Fcrl5*; hence, the supplementation with SCFAs might regulate immune-related transcripts in the midgut of grass carp. Simultaneously, *Itgb2*, as a lymphoid and megakaryocytic lineage marker protein, plays important roles in protecting fish from a variety of pathogens (50). It was mentioned above that *Itgb2* was negatively correlated with intestinal-related bacteria *Rikenellaceae_RC9_gut_group* and not-assigned *Intestinimonas*. Previous study had shown that *Rikenellaceae_RC9_gut_group* in high-fat diet-fed mice might be positively associated with lipopolysaccharide, which is considered as one main trigger for chronic inflammation and destroy the intestinal barrier (51). Therefore, compared with the control group, we speculated that the significant decrease of *Itgb2* resulted from the florfenicol treatment (**Figure 5J**) helped increase the levels of some LPS-producing bacterial genera, subsequently damage to the physical gut barrier.

Tight junction proteins—widely existing at the top of the junction between intestinal epithelial cells—are important to intestinal homeostasis because intestinal epithelial cells form an integrated whole *via* tight junctions to maintain their own polarity (52, 53). One study reported that a broad-spectrum antibiotic cocktail was able to induce intestinal tight junction barrier dysfunction by activating the NLRP3 inflammasome and autophagy in healthy mice (54). That finding is consistent with our results of significantly lower expression levels of *Claudin6* and *ZO1* in grass carp exposed to florfenicol (**Figure 7A**). Intestinal microbiota play important roles in the safeguarding the integrity and functioning of the mucosal barrier and they should be targeted when evaluating intestinal health (55). Our correlation analysis of key genera with mucosal barrier proteins showed that *Cetobacterium* had a positive correlation with *ZO1* and *Claudin6*, while *ZOR0006* had a positive correlation with *ZO3* and *Claudin6*, thus indicating that these key genera contribute to the intestine mucosal barrier function of grass carp fish.

Goblet cells, secreted in the mucus layer overlying the epithelium, figure prominently in the clearance of intestinal contents to guard against physical-chemical damages caused by microorganisms and microbial attachments (56). Sun, Korheina (57) showed that the number of goblet cells in the intestine was suppressed by the chronic exposure to dietary antibiotics in the Oriental River prawn (*Macrobrachium nipponense*). In the present study, enrofloxacin and florfenicol reduced the number of goblet cells (**Figure 8**), likely impairing their normal functioning. These observations suggest that antibiotics can reduce the permeability of intestinal epithelial cells, induce the apoptosis, and thereby possibly impair host fish immunity.

Apoptosis, or programmed cell death, is autonomous and regulated by phagocytosis of cellular remnants (58, 59). Work by Liu, Cui (60) demonstrated that enrofloxacin induces apoptosis in the hepatic cell line of grass carp. Later, Qiu, Hu (9) confirmed that exposure to antibiotics at environmentally relevant concentrations increased apoptosis in primary macrophages of common carp. Those observations *in vivo* are consistent with our findings that both enrofloxacin and florfenicol triggered apoptosis in grass carp *in vitro* (**Figure 9**).

In conclusion, this study provides a timely understanding of the substantial effects of enrofloxacin and florfenicol oral treatments on the immune response, gut microbiota, and transcriptome profiles of healthy grass carp. A 14-day antibiotic treatment (enrofloxacin or florfenicol) can impact the intestinal morphology and mucosal immune system. Enrofloxacin but not florfenicol changes the gut microbiota diversity in grass carp. Compared with enrofloxacin, florfenicol relatively altered gut transcriptome profiles in grass carp. Both antibiotics, however, can induce apoptosis in the midguts of grass carp. Arguably, for these reasons, the use of enrofloxacin and florfenicol should be effectively banned to maintain fish health in aquaculture.

## Data availability statement

The data presented in the study are deposited in the ArrayExpress repository (https://www.ebi.ac.uk/arrayexpress), the accession number E-MTAB-12057 and E-MTAB-12058 for 16S rDNA high-throughput and transcriptomic sequencing, respectively.

## Ethics statement

The animal study was reviewed and approved by the Animal Ethics Committee of Zhongkai University of Agriculture and Engineering.

## Author contributions

FS and YH performed experiments, analyzed the data, and wrote the manuscript. MY, ZL, YL, and FZ performed the experiments. LL and ZQ conceived ideas, analyzed the data, oversaw the research, and wrote the manuscript. All authors contributed to the article and approved the submitted version.

## Funding

This research was jointly supported by the National Natural Science Foundation of China (4210061024), and Open Foundation of Key Laboratory of Tropical Marine Bio-resources and Ecology, Chinese Academy of Sciences (LMB20211004).

## Conflict of interest

The authors declare that the research was conducted in the absence of any commercial or financial relationships that could be construed as a potential conflict of interest.

## Publisher’s note

All claims expressed in this article are solely those of the authors and do not necessarily represent those of their affiliated organizations, or those of the publisher, the editors and the reviewers. Any product that may be evaluated in this article, or claim that may be made by its manufacturer, is not guaranteed or endorsed by the publisher.
